# An artificial neural network model for evaluating the risk of hyperuricaemia in type 2 diabetes mellitus

**DOI:** 10.1038/s41598-024-52550-1

**Published:** 2024-01-25

**Authors:** Qingquan Chen, Haiping Hu, Yuanyu She, Qing He, Xinfeng Huang, Huanhuan Shi, Xiangyu Cao, Xiaoyang Zhang, Youqiong Xu

**Affiliations:** 1 The Affiliated Fuzhou Center for Disease Control and Prevention of Fujian Medical University, Fuzhou, China; 2 School of Public Health, Fujian Medical University, Fuzhou, China

**Keywords:** Medical research, Biomarkers, Epidemiology, Endocrine system and metabolic diseases, Diabetes

## Abstract

Type 2 diabetes with hyperuricaemia may lead to gout, kidney damage, hypertension, coronary heart disease, etc., further aggravating the condition of diabetes as well as adding to the medical and financial burden. To construct a risk model for hyperuricaemia in patients with type 2 diabetes mellitus based on artificial neural network, and to evaluate the effectiveness of the risk model to provide directions for the prevention and control of the disease in this population. From June to December 2022, 8243 patients with type 2 diabetes were recruited from six community service centers for questionnaire and physical examination. Secondly, the collected data were used to select suitable variables and based on the comparison results, logistic regression was used to screen the variable characteristics. Finally, three risk models for evaluating the risk of hyperuricaemia in type 2 diabetes mellitus were developed using an artificial neural network algorithm and evaluated for performance. A total of eleven factors affecting the development of hyperuricaemia in patients with type 2 diabetes mellitus in this study, including gender, waist circumference, diabetes medication use, diastolic blood pressure, γ-glutamyl transferase, blood urea nitrogen, triglycerides, low-density lipoprotein cholesterol, high-density lipoprotein cholesterol, fasting glucose and estimated glomerular filtration rate. Among the generated models, baseline & biochemical risk model had the best performance with cutoff, area under the curve, accuracy, recall, specificity, positive likelihood ratio, negative likelihood ratio, precision, negative predictive value, KAPPA and F1-score were 0.488, 0.744, 0.689, 0.625, 0.749, 2.489, 0.501, 0.697, 0.684, 0.375 and 0.659. In addition, its Brier score was 0.169 and the calibration curve also showed good agreement between fitting and observation. The constructed artificial neural network model has better efficacy and facilitates the reduction of the harm caused by type 2 diabetes mellitus combined with hyperuricaemia.

## Introduction

Type 2 diabetes is a chronic metabolic disease caused by a combination of genetic, dietary and environmental factors. The disease is characterised by insufficient insulin secretion or an inability to utilise insulin efficiently, resulting in persistent elevation of blood glucose^1^. According to a report by the World Health Organization (WHO), diabetes is the direct cause of 1.5 million deaths in 2019, with 48% of diabetes deaths occurring before the age of 70^2^. According to the data released by the International Diabetes Federation (IDF) in 2021, there are 140 million people with diabetes in China, with a prevalence rate of approximately 10.6%, and both the number of people with the disease and the prevalence rate are on the rise, with type 2 diabetes accounting for more than 90% of the Chinese diabetic population^3^. In addition, diabetes mellitus may lead to various complications, such as blindness^4^, kidney failure^5^ and hypertension^6^, due to factors such as poor blood sugar control over a long period of time.

Hyperuricaemia is the greatest risk factor for gout^7^ and is mainly due to excessive production or poor excretion of uric acid, the main source of which is purines^8^. Past studies have shown that hyperuricaemia is a risk factor for diabetes mellitus, cardiovascular disease, metabolic syndrome and other diseases^9–11^. A meta-analysis showed that the prevalence of hyperuricaemia in the Chinese population was 16.4%^12^.

Studies have shown that type 2 diabetes and hyperuricaemia can interact. On the one hand, people with type 2 diabetes are often insulin resistant, which may lead to increased tubular reabsorption of uric acid, which can lead to hyperuricaemia^13,14^. On the other hand, epidemiological studies have shown that hyperuricaemia is a risk factor for insulin resistance, prediabetes and diabetes^9,11,15^. In addition, recent evidence suggests that high levels of uric acid interfere with insulin signalling in endothelial cells at both the receptor and post-receptor levels, and that at the post-receptor level, both proximal (IRS and PI3K-Akt components) and distal (eNOS-NO system) steps of the insulin signalling pathway are affected by uric acid^16^. Risk predictors of high uric acid levels in patients with type 2 diabetes mellitus have been explored, including hip circumference, total cholesterol, high-density lipoprotein, etc.^17,18^.

Previous studies have applied the Cox regression model and machine learning methods to build a risk model of hyperuricemia based on sociodemographic data, routine physical examination markers, dietary risk factors, blood biomarkers, and alterations of the gut microbiome^19–24^. However, these established studies have only modelled hyperuricaemia in healthy population, while there was a study exploring the development of a hyperuricemia risk model in diabetic kidney disease patients^25^. To the best of our knowledge, only one study has established a predictive model of hyperuricaemia in the type 2 diabetic population^26^.

In recent years, Artificial neural networks (ANN) have become popular and useful models for classification, clustering, pattern recognition and prediction in many disciplines. It has a fast and wide range of uses in dealing with a variety of complex real-world problems^27^. The popularity of ANN lies in its information processing characteristics, including learning ability, high parallelism, fault tolerance, nonlinearity, noise tolerance and generalisation^27,28^. Also, Dalakleidi K et al.^29^ showed, ANN is superior to other machine learning algorithms. Although, ANN advantages are obvious, the previous did not use ANN algorithm to model the risk of hyperuricaemia risk factors.

In this study, we constructed a risk model for hyperuricaemia in patients with type 2 diabetes mellitus based on ANN algorithm, and assessed the validity of the model. This has an important role in clinically distinguishing high-risk individuals and identifying risk factors, which in turn has far-reaching significance in alleviating disease symptoms, reducing the risk of patient death and reducing the healthcare burden.

## Methods

### Study participants

This was a retrospective cross-sectional survey. Between June and December 2022, we randomly recruited patients with type 2 diabetes from one community in each of the six urban areas of Fuzhou City. All participants underwent a face-to-face survey using a homemade uniform questionnaire and took a physical examination, which were both conducted by trained primary care professionals.

Patients with malignancy, history of gout, hyperuricaemia occurring before type 2 diabetes mellitus, type 1 diabetes mellitus, gestational diabetes mellitus and other specific diabetes mellitus were not included in this study. After exclusion of incomplete physical examination data, a total of 8243 cases were obtained.

All respondents completed an informed consent form the ethical research board committee of Fuzhou Center for Disease Control and Prevention (approval number: 2022002) approved the research. In addition, all participants and/or their legal guardians consented to use their medical data in this study. This study was carried out following the Helsinki Declaration contents.

### Data measurements

Basic personal information and medical history were investigated through questionnaires, including gender, age, history of smoking and alcohol consumption, duration of diabetes, medication history, etc.

Physical examination was performed to obtain data covering height, weight, waist circumference, blood pressure, etc.

Biochemical indicators were obtained through laboratory tests. Patients were fasted overnight for at least 10 h and drank water for at least 8 h, and fasting venous blood was taken between 7:00 and 9:00 am the following morning. Fasting blood glucose (FPG), uric acid (UA), alanine aminotransferase (ALT), total bilirubin (TBil), serum creatinine (SCr), γ-glutamyl transpeptidase (GGT), blood urea nitrogen (BUN), total cholesterol (TC), triglycerides (TGs), low-density lipoprotein cholesterol (LDL-C) and high-density lipoprotein cholesterol (HDL-C) were measured using a fully automated biochemistry analyser (Model 7100, Hitachi, Japan).

### Description of variables

**Figure 1:**
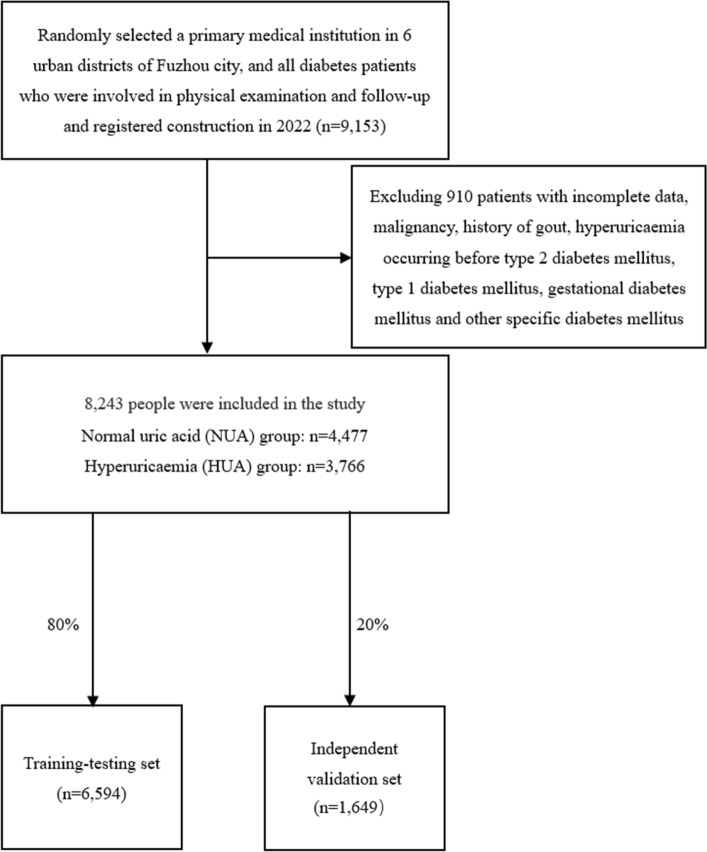
Flow chart of the study.

1. Men with uric acid < 420 μmol/L and women with uric acid < 360 μmol/L were considered the normal uric acid group, with a total of 4,477 cases; men with uric acid > 420 μmol/L and women with uric acid > 360 μmol/L were considered the high uric acid group, with a total of 3,766 cases (Fig. 1).
2. According to the Chinese Comprehensive Diabetes Control Objectives (2019)^30^, the normal reference range: FPG:4.4 to 7.0 mmol/L; blood pressure: < 130/80 mm Hg; TC: < 4.5 mmol/L; TGs: < 1.7 mmol/L; LDL-C: < 2.6 mmol/L (uncomplicated atherosclerotic cardiovascular disease) or < 1.8 mmol/L (complicated atherosclerotic cardiovascular disease); HDL-C: > 1.0 mmol/L (men) or > 1.3 mmol/L (women); uric acid: upper limit < 420 μmol/L for men and < 360 μmol/L for women; SCr: 55–133 μmol/L for men and 44–97 μmol/L for women; BUN: 2.9–7.5 mmol/L; ALT: 5 to 40 U/L; GGT: < 40U/L; TBil: 1.71 to 17.10 μmol/L; waist circumference ≥ 90 cm for men and ≥ 85 cm for women as central obesity; body mass index (BMI) < 18.5 kg/m^2^ is considered underweight, normal reference range 18.5 kg/m^2^ ≤ BMI < 24.0 kg/m^2^, BMI ≥ 24.0 kg/m^2^ is considered overweight, BMI ≥ 28.0 kg/m^2^ is considered obese. BMI = weight/height^2^.
3. Estimation of glomerular filtration rate (eGFR): eGFR was calculated using the Chronic Kidney Disease Epidemiology Cooperative Study Group (CKD-EPI) formula^31^: for men with SCr ≤ 0.9 mg/dl, eGFR = 141 × (SCr/0.9)^-0.411^ × 0.993^age^; Female SCr ≤ 0.7 mg/dl, eGFR = 144 × (SCr/0.7)^-0.329^ × 0.993^age^; male Scr > 0.9 ml/dl: eGFR = 144 × (Scr/0.9)^-1.209^ × (0.993)^age^; female SCr > 0.7 mg/dl, eGFR = 141 × (SCr/0.7)^-1.209^ × 0.993^age^. The Chinese guidelines for the prevention and treatment of type 2 diabetes mellitus define eGFR < 60 mL/min × 1.73m^2^ as a decrease in the GFR^32^.
4. WHO defines smokers as "those who have smoked continuously or cumulatively for ≥ 6 months in their lifetime"; alcohol drinkers are defined as those who have consumed alcohol at least once a week for ≥ 6 months; and adequate exercise is defined as achieving moderate intensity exercise, with a duration of ≥ 30 min per exercise session and frequency ≥ 3 times per week.

### Statistical methods

Data were double entered using EpiData (version 3.1) and analysed using IBM SPSS (version 22.0) and RStudio (version 4.2.3); measured data conforming to a normal distribution were expressed as ($\overline{x }$±*s*) and compared between groups by a *t* test, and count data were compared by a chi-square test. Univariate and multivariate logistic regression analyses were conducted using uric acid levels as a dependent variable and sociodemographic characteristics and physiological and biochemical indicators as independent variables, with variables introduced and excluded at a test level of 0.05. The variance inflation factor (VIF) was used to examine collinearity among the independent variables included in the multivariate logistic regression analysis in this study. Data management and statistical analysis were conducted using R version 4.3.2.

### Development and validation of the classification models

We utilized multivariable stepwise logistic regression analysis for variable selection. The ANN algorithm was used to build models for three different data scenarios (baseline data only, biochemical indicators only, and baseline data and biochemical indicators).

The incorporated data were divided into a training–testing set (80%) and an independent validation set (20%) using stratified sampling. We utilized grid search to search the hyperparameter space efficiently. This allowed us to find the optimal combination of hyperparameters for three ANN models. To avoid overfitting and promote the models, we used a tenfold cross-validation for the training–testing set and referenced the best models to the independent validation set.

**Figure 2:**
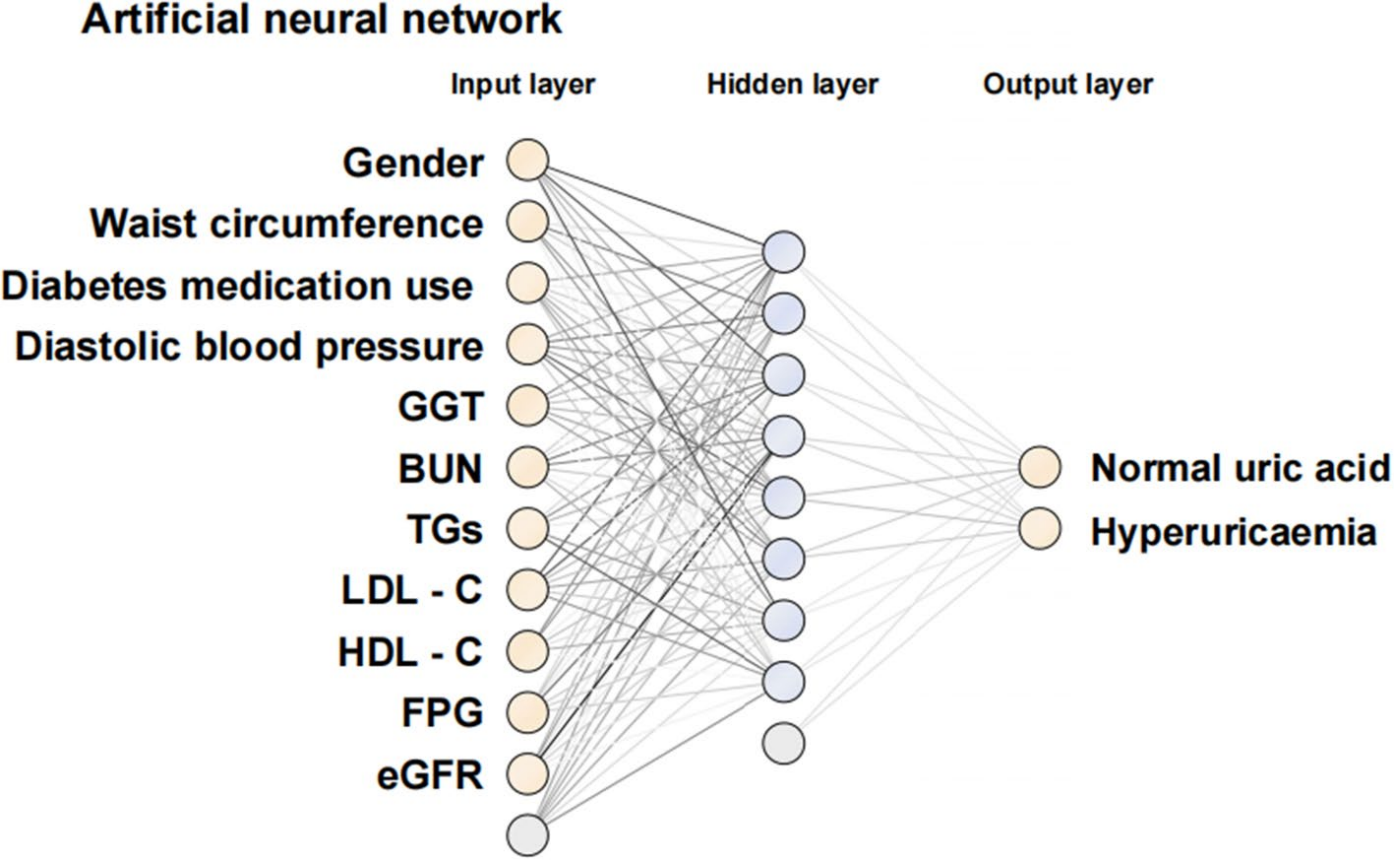
Artificial neural network model.

The areas under curves (AUCs) of the three ANN models in the training–testing set were evaluated to assess model performance. In addition, we calculated performance metrics including AUC, accuracy, recall, specificity, positive likelihood ratio (PLR), negative likelihood ratio (NLR), precision, negative predictive value (NPV), kappa, and F1-score. After a comparison of the above performance metrics, the constructed optimal ANN model is visible in Fig. 2. Finally, the calibration curve was analyzed to assess the agreement by the slope, intercept, and Brier score (an ideal value of 0; a value of > 0.3 indicates poor calibration) of the calibration curve.

All models were performed using R version 4.3.2.

### Ethics approval and consent

This study was approved by the Ethics Committee of the Fuzhou Center for Disease Control and Prevention (approval number: 2022002). Informed consent was obtained from all participants and/or their legal guardians for this study. There is no conflict of interest in this study.

## Results

### Demographic characteristics

A total of 8243 diabetic patients were investigated in this survey. Table 1 shows descriptive statistics of sample characteristics, including age, gender, tobacco use, drinking alcohol, sport, waist circumference, BMI, disease duration, diabetes medication use, systolic blood pressure and diastolic blood pressure.

**Table 1:** Demographic characteristics of the patients in this survey (N = 8243).

Characteristic	Contents	Participants, n (%)
Age	< 65	2226 (27.0)
	≥ 65	6017 (73.0)
Gender	Male	3588 (43.5)
	Female	4655 (56.5)
Tobacco use	No	7211 (87.5)
	Yes	1032 (12.5)
Drinking alcohol	No	7234 (87.8)
	Yes	1009 (12.2)
Sport	Adequate	6017 (73.0)
	Lacking	2226 (27.0)
Waist circumference(cm)	Male < 90 / Female < 85	4326 (52.5)
	Male ≥ 90 / Female ≥ 85	3917 (47.5)
BMI (kg/m^2^)	< 24.0	3408 (41.3)
	≥ 24.0	4835 (58.7)
Disease duration (years)	< 10	4710 (57.1)
	≥ 10	3533 (42.9)
Diabetes medication use^a^	Use	7214 (87.5)
	No	1029 (12.5)
SBP (mm Hg)	< 130	3224 (39.1)
	≥ 130	5019 (60.9)
DBP (mm Hg)	< 80	4270 (51.8)
	≥ 80	3973 (48.2)

BMI: body mass index; SBP: systolic blood pressure; DBP: diastolic blood pressure; a: Based on the results of the questionnaire.

### Univariate and multivariate analyses of baseline information

Baseline data were included in separate univariate logistic regression analyses to screen for a total of eight variables: gender, tobacco use, alcohol use, exercise, waist circumference, BMI, diabetes medication use and DBP (*P* < 0.05). The VIF for these eight baseline variables are all less than 5, so there is no multicollinearity (Table 5). Further inclusion in the multivariate logistic regression analysis revealed that gender, exercise, waist circumference, DBP and diabetic medication use were influential factors (*P* < 0.05). Details are presented in Table 2.

**Table 2:** Univariate and multivariate logistic regression analysis of baseline information.

Variables	N = 8243	Uric acid status		Univariate analysis			Multivariate analysis	
		NUA group	HUA group	χ^2^	Degrees of freedom	*p*	OR (95%*CI*)	*p*
Age								
< 65^R^	2226	1195(26.7)	1031(27.4)	0.452	1	0.501		
≥ 65	6017	3282(73.3)	2735(72.6)					
Gender								
Male^R^	3588	2613(58.4)	975(25.9)	876.270	1	< 0.001	4.09[3.67,4.57]	< 0.001
Female	4655	1864(41.6)	2791(74.1)					
Tobacco use								
No^R^	7211	3732(83.4)	3479(92.4)	151.120	1	< 0.001	0.98[0.82,1.16]	0.776
Yes	1032	745(16.6)	287(7.6)					
Drinking alcohol								
No^R^	7234	3779(84.4)	3455(91.7)	101.700	1	< 0.001	1.06[0.90,1.26]	0.467
Yes	1009	698(15.6)	311(8.3)					
Sport								
Adequate^R^	6017	3353(74.9)	2664(70.7)	17.710	1	< 0.001	1.13[1.02,1.25]	0.024
Lacking	2226	1124(25.1)	1102(29.3)					
Waist circumference(cm)								
Male < 90 /Female < 85^R^	4326	2602(58.1)	1724(45.8)	124.430	1	< 0.001	1.45[1.30,1.63]	< 0.001
Male ≥ 90/Female ≥ 85	3917	1875(41.9)	2042(54.2)					
BMI (kg/m^2^)								
< 24.0^R^	3408	1996(44.6)	1412(37.5)	42.107	1	< 0.001	1.08[0.96,1.21]	0.177
≥ 24.0	4835	2481(55.4)	2354(62.5)					
Disease duration (years)								
< 10^R^	4710	2544(56.8)	2166(57.5)	0.371	1	0.543		
≥ 10	3533	1933(43.2)	1600(42.5)					
Diabetes medication use								
Use^R^	7214	3864(86.3)	3350(89.0)	12.867	1	< 0.001	0.74[0.64,0.85]	< 0.001
No	1029	613(13.7)	416(11.0)					
SBP (mm Hg)								
< 130^R^	3224	1792(40.0)	1432(38.0)	3.360	1	0.067		
≥ 130	5019	2685(60.0)	2334(62.0)					
DBP (mm Hg)								
< 80^R^	4270	2420(54.1)	1850(49.1)	19.717	1	< 0.001	1.27[1.16,1.40]	< 0.001
≥ 80	3973	2057(45.9)	1916(50.9)					

R: reference, BMI: body mass index, SBP: systolic blood pressure, DBP: diastolic blood pressure, CI: confidence interval.

### Univariate and multivariate analyses of biochemical indicators

Biochemical indicators were included in the univariate logistic regression analysis, and a total of nine variables, including GGT, TBil, BUN, TGs, TC, LDL-C, HDL-C, FPG and the eGFR, were screened (*P* < 0.05). There is no multicollinearity among nine biochemical indicators variables (all VIF < 5) (Table 5). Further inclusion in the multivariate logistic regression analysis revealed that GGT, TBil, BUN, TGs, TC, LDL-C, HDL-C, FPG and the eGFR were influential factors for hyperuricaemia (*P* < 0.05, Table 3).

**Table 3:** Univariate and multivariate logistic regression analysis of biochemical indicators.

Variables	N = 8243	Uric acid status		Univariate analysis			Multivariate analysis	
		NUA group	HUA group	χ^2^	Degrees of freedom	*p*	OR (95%*CI*)	*p*
ALT (U/L)								
< 40^R^	7292	3981(88.9)	3311(87.9)	1.919	1	0.166		
≥ 40	951	496(11.1)	455(12.1)					
GGT (U/L)								
< 40^R^	7765	4258(95.1)	3507(93.1)	14.402	1	< 0.001	1.48[1.22,1.79]	< 0.001
≥ 40	478	219(4.9)	259(6.9)					
TBil (μmol/L)								
< 17.1^R^	7064	3749(83.7)	3315(88.0)	30.296	1	< 0.001	0.79[0.69,0.90]	< 0.001
≥ 17.1	1179	728(16.3)	451(12.0)					
BUN (mmol/L)								
< 7.5^R^	7643	4213(94.1)	3430(91.1)	27.288	1	< 0.001	1.22[1.01,1.47]	0.041
≥ 7.5	600	264(5.9)	336(8.9)					
TGs (mmol/L)								
< 1.7^R^	2447	1447(32.3)	1000(26.6)	175.420	1	< 0.001	1.55[1.41,1.70]	< 0.001
≥ 1.7	5796	3030(67.7)	2766(73.4)					
TC (mmol/L)								
< 4.5^R^	4519	2753(61.5)	1766(46.9)	32.319	1	< 0.001	1.18[1.05,1.33]	0.001
≥ 4.5	3724	1724(38.5)	2000(53.1)					
LDL—C (mmol/L)								
< 2.6^R^	2757	1595(35.6)	1162(30.9)	20.707	1	< 0.001	1.18[1.05,1.33]	0.005
≥ 2.6	5486	2882(64.4)	2604(69.1)					
HDL—C (mmol/L)								
Male > 1.0/Female > 1.3^R^	5745	3400(75.9)	2345(62.3)	180.480	1	< 0.001	1.83[1.65,2.02]	< 0.001
Male ≤ 1.0/Female ≤ 1.3	2498	1077(24.1)	1421(37.7)					
FPG (mmol/L)								
< 7.0^R^	4529	2403(53.7)	2126(56.5)	6.265	1	0.012	0.81[0.74,0.88]	< 0.001
≥ 7.0	3714	2074(46.3)	1640(43.5)					
eGFR (mL/min × 1.73m^2^)								
≥ 60^R^	613	204(4.6)	409(10.9)	117.160	1	< 0.001	2.24[1.85,2.72]	< 0.001
< 60	7630	4273(95.4)	3357(89.1)					

R: reference, ALT: alanine aminotransferase, GGT: γ-glutamyl transpeptidase, TBil: total bilirubin, BUN: blood urea nitrogen, TGs: triglycerides, TC: total cholesterol, LDL-C: low-density lipoprotein cholesterol, HDL-C: high-density lipoprotein cholesterol, FPG: fasting glucose, eGFR: estimated glomerular filtration rate. CI: confidence interval. The eGFR was selected in two similar variables, eGFR and SCr.

### Univariate and multivariate analyses of baseline & biochemical indicators

Baseline and biochemical indicators were included in the univariate logistic regression analysis, and a total of seventeen variables, including gender, tobacco use, alcohol use, exercise, waist circumference, BMI, diabetes medication use, DBP, GGT, TBil, BUN, TGs, TC, LDL-C, HDL-C, FPG and the eGFR, were screened (*P* < 0.05). There is no multicollinearity among 17 baseline & biochemical indicators variables (all VIF < 5) (Table 5). Further inclusion in the multivariate logistic regression analysis revealed that gender, waist circumference, diabetes medication use, DBP, GGT, BUN, TGs, LDL-C, HDL-C, FPG and the eGFR were influential factors for hyperuricaemia (*P* < 0.05) (Table 4).

**Table 4:** Univariate and multivariate logistic regression analysis of baseline & biochemical indicators.

Variables	N = 8243	Uric acid status		Univariate analysis			Multivariate analysis	
		NUA group	HUA group	χ^2^	Degrees of freedom	*p*	OR (95%CI)	*p*
Age								
< 65^R^	2226	1195(26.7)	1031(27.4)	0.452	1	0.501		
≥ 65	6017	3282(73.3)	2735(72.6)					
Gender								
Male^R^	3588	2613(58.4)	975(25.9)	876.270	1	< 0.001	4.15[3.70,4.66]	< 0.001
Female	4655	1864(41.6)	2791(74.1)					
Tobacco use								
No^R^	7211	3732(83.4)	3479(92.4)	151.120	1	< 0.001	0.94[0.78,1.12]	0.480
Yes	1032	745(16.6)	287(7.6)					
Drinking alcohol								
No^R^	7234	3779(84.4)	3455(91.7)	101.700	1	< 0.001	1.10[0.93,1.31]	0.261
Yes	1009	698(15.6)	311(8.3)					
Sport								
Adequate^R^	6017	3353(74.9)	2664(70.7)	17.710	1	< 0.001	1.08[0.97,1.20]	0.165
Lacking	2226	1124(25.1)	1102(29.3)					
Waist circumference (cm)								
Male < 90/Female < 85^R^	4326	2602(58.1)	1724(45.8)	124.430	1	< 0.001	1.38[1.23,1.55]	< 0.001
Male ≥ 90/Female ≥ 85	3917	1875(41.9)	2042(54.2)					
BMI (kg/m^2^)								
< 24.0^R^	3408	1996(44.6)	1412(37.5)	42.107	1	< 0.001	1.04[0.93,1.17]	0.464
≥ 24.0	4835	2481(55.4)	2354(62.5)					
Disease duration (years)								
< 10^R^	4710	2544(56.8)	2166(57.5)	0.371	1	0.543		
≥ 10	3533	1933(43.2)	1600(42.5)					
Diabetes medication use								
Use^R^	7214	3864(86.3)	3350(89.0)	12.867	1	< 0.001	0.75[0.65,0.86]	< 0.001
No	1029	613(13.7)	416(11.0)					
SBP (mm Hg)								
< 130^R^	3224	1792(40.0)	1432(38.0)	3.360	1	0.067		
≥ 130	5019	2685(60.0)	2334(62.0)					
DBP (mm Hg)								
< 80^R^	4270	2420(54.1)	1850(49.1)	19.717	1	< 0.001	1.30[1.18,1.44]	< 0.001
≥ 80	3973	2057(45.9)	1916(50.9)					
ALT (U/L)								
< 40^R^	7292	3981(88.9)	3311(87.9)	1.919	1	0.166		
≥ 40	951	496(11.1)	455(12.1)					
GGT (U/L)								
< 40^R^	7765	4258(95.1)	3507(93.1)	14.402	1	< 0.001	1.38[1.13,1.70]	0.002
≥ 40	478	219(4.9)	259(6.9)					
TBil (μmol/L)								
< 17.1^R^	7064	3749(83.7)	3315(88.0)	30.296	1	< 0.001	0.97[0.85,1.12]	0.711
≥ 17.1	1179	728(16.3)	451(12.0)					
BUN (mmol/L)								
< 7.5^R^	7643	4213(94.1)	3430(91.1)	27.288	1	< 0.001	1.42[1.16,1.74]	0.001
≥ 7.5	600	264(5.9)	336(8.9)					
TGs (mmol/L)								
< 1.7^R^	2447	1447(32.3)	1000(26.6)	175.420	1	< 0.001	1.71[1.54,1.89]	< 0.001
≥ 1.7	5796	3030(67.7)	2766(73.4)					
TC (mmol/L)								
< 4.5^R^	4519	2753(61.5)	1766(46.9)	32.319	1	< 0.001	0.99[0.87,1.12]	0.835
≥ 4.5	3724	1724(38.5)	2000(53.1)					
LDL—C (mmol/L)								
< 2.6^R^	2757	1595(35.6)	1162(30.9)	20.707	1	< 0.001	1.13[1.00,1.28]	0.046
≥ 2.6	5486	2882(64.4)	2604(69.1)					
HDL—C (mmol/L)								
Male > 1.0/Female > 1.3^R^	5745	3400(75.9)	2345(62.3)	180.480	1	< 0.001	1.23[1.10,1.37]	< 0.001
Male ≤ 1.0/Female ≤ 1.3	2498	1077(24.1)	1421(37.7)					
FPG (mmol/L)								
< 7.0^R^	4529	2403(53.7)	2126(56.5)	6.265	1	0.012	0.77[0.70,0.85]	< 0.001
≥ 7.0	3714	2074(46.3)	1640(43.5)					
eGFR (mL/min × 1.73m^2^)								
≥ 60^R^	613	204(4.6)	409(10.9)	117.160	1	< 0.001	2.59[2.12,3.19]	< 0.001
< 60	7630	4273(95.4)	3357(89.1)					

R: reference, SBP: systolic blood pressure, DBP: diastolic blood pressure, ALT: alanine aminotransferase, GGT: γ-glutamyl transpeptidase, TBil: total bilirubin, BUN: blood urea nitrogen, TGs: triglycerides, TC: total cholesterol, LDL-C: low-density lipoprotein cholesterol, HDL-C: high-density lipoprotein cholesterol, FPG: fasting glucose, eGFR: estimated glomerular filtration rate, CI: confidence interval.

### Model performance

**Figure 3:**
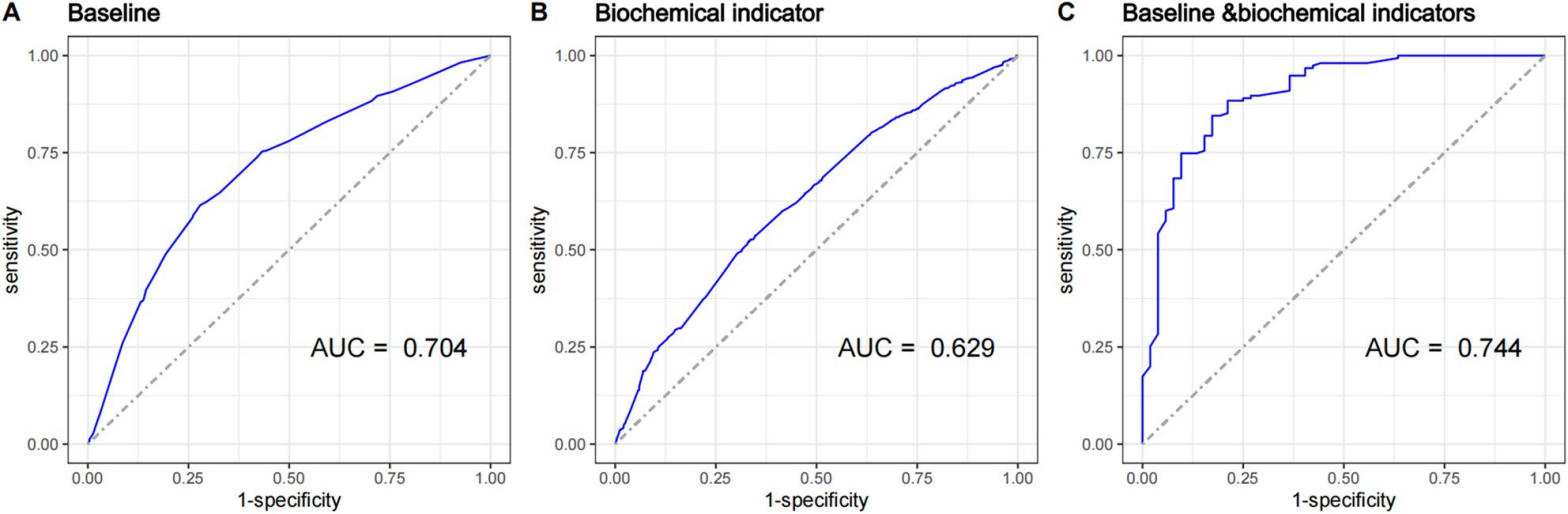
Areas under curves (AUCs) of the three ANN models developed.

**Figure 4:**
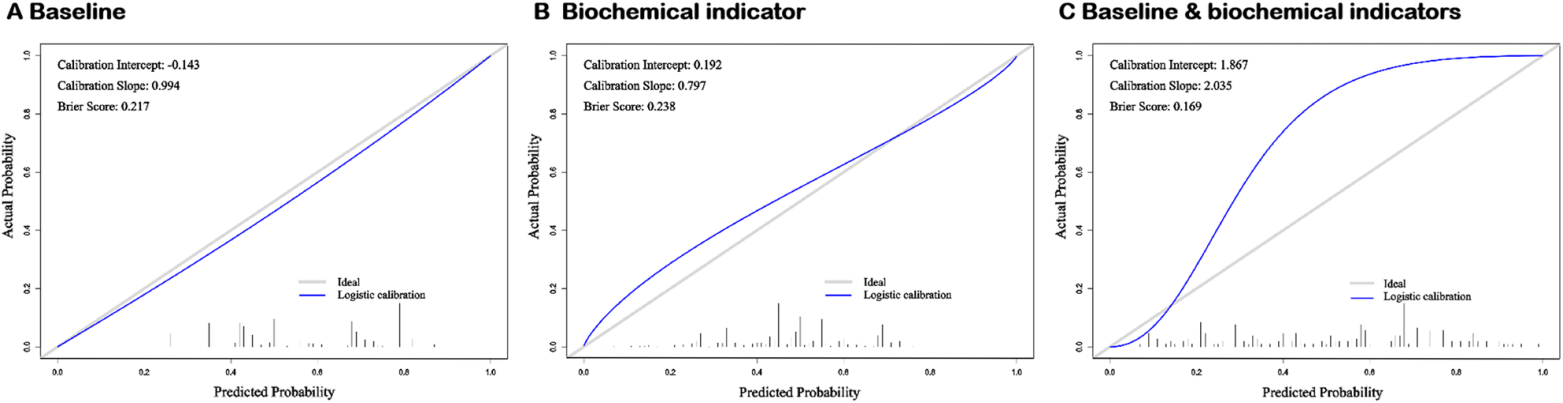
Calibration curves for testing the stability of three models in the study.

As described previously, after certain inclusion and exclusion criteria, we built three models, with baseline data, biochemical indicators and baseline & biochemical indicators, respectively (Table 5). By using Grid-optimization**,** the hyperparameters of the optimal ANN model were : hidden = c(8), threshold = 0.01, stepmax = 1e + 05, rep = 1, learningrate.factor = list(minus = 0.5, plus = 1.2), lifesign.step = 1000, algorithm = rprop + , err.fct = sse, act.fct = logistic. Figure 3 and Table 6 show the performances of all three models. After comparison, Baseline & biochemical model has the best performance with cutoff, AUC, accuracy, recall, specificity, PLR, NLR, precision, NPV, KAPPA and F1-score were 0.488, 0.744(0.721–0.768), 0.689(0.689**–**0.690), 0.625(0.591–0.658), 0.749(0.720–0.778), 2.489(2.191–2.828), 0.501(0.454–0.553), 0.697(0.663–0.731), 0.684(0.654–0.713), 0.375(0.331–0.420) and 0.659(0.625–0.693). In addition, its Brier score was 0.169 and the calibration curve also showed good agreement between fitting and observation (Fig. 4).

**Table 5:** Colinearity diagnostics of independent variables included in the above three multivariate logistic regression analysis in this study.

Variables	Baseline	Biochemical indicator	Baseline and biochemical indicators
Gender	1.288	NA	1.410
Waist circumference	1.476	NA	1.495
DBP	1.028	NA	1.042
BMI	1.479	NA	1.490
Diabetes medication use	1.003	NA	1.032
Tobacco use	1.382	NA	1.391
Drinking alcohol	1.322	NA	1.330
Sport	1.008	NA	1.014
GGT	NA	1.012	1.017
TBil	NA	1.023	1.047
BUN	NA	1.180	1.183
TC	NA	1.524	1.560
TGs	NA	1.107	1.125
LDL—C	NA	1.500	1.507
HDL—C	NA	1.105	1.190
FPG	NA	1.016	1.047
eGFR	NA	1.194	1.203

**Table 6:** Performance comparison of the three models developed.

Characteristic	Baseline	Biochemical indicator	Baseline and biochemical indicators
Cutoff	0.588	0.516	0.488
AUC (95% *CI*)	0.704(0.679–0.730)	0.629(0.602–0.655)	0.744(0.721–0.768)
Accuracy (95% *CI*)	0.663(0.663–0.663)	0.589(0.589–0.589)	0.689(0.690–0.689)
Recall (95% *CI*)	0.615(0.583–0.646)	0.526(0.494–0.559)	0.625(0.591–0.658)
Specificity (95% *CI*)	0.721(0.689–0.753)	0.664(0.630–0.698)	0.749(0.720–0.778)
PLR (95% *CI*)	2.204(1.943–2.499)	1.566(1.392–1.763)	2.489(2.191–2.828)
NLR (95% *CI*)	0.535(0.487–0.587)	0.713(0.655–0.777)	0.501(0.454–0.553)
Precision (95% *CI*)	0.724(0.692–0.755)	0.651(0.616–0.685)	0.697(0.663–0.731)
NPV (95% *CI*)	0.611(0.579–0.644)	0.541(0.509–0.573)	0.684(0.654–0.713)
KAPPA (95% *CI*)	0.331(0.286–0.376)	0.187(0.141–0.233)	0.375(0.331–0.420)
F1-score (95% *CI*)	0.665(0.633–0.696)	0.582(0.548–0.616)	0.659(0.625–0.693)

CI: confidence interval, AUC: area under the curve, PLR: positive likelihood ratio, NLR: negative likelihood ratio, NPV: negative predictive value.

## Discussion

Although ANN have been widely used in predictive modelling of diseases, however, as far as we know, no study has modelled the risk of hyperuricaemia in a large sample of type 2 diabetic population, as previous studies have only modelled and analysed type 2 diabetes^33,34^ or hyperuricaemia^19^. After comparing the performances of the three models and after model validation, we confirmed that the baseline and biochemical model was the optimal model. Interestingly, we noted in our study that the model for baseline information was superior to that for biochemical indicators. Baseline information is relatively stable and more reflective of the patient's true condition over a long period of time than biochemical indicators, which are one-off test results that only indicate the status of the patient's biochemical levels for the first day or two or the first few days of testing. In other words, in a cross-sectional study, baseline information may be more important and more reflective of the patient's true condition than biochemical indicators. Certainly, we will need to demonstrate this in future studies.

We have long identified hyperuricaemia as the greatest risk factor for gout^7^, and men are usually more likely to develop hyperuricaemia than premenopausal women^35^. However, our study showed that the detection rate of hyperuricaemia was not only higher in women (33.86%) than in men (11.83%) in the type 2 diabetic population but also the risk of developing hyperuricaemia was 4.15 times higher (95%*Cl* 3.70–4.66) in women than in men; we also noted that the confidence interval for the mean age of women in this population was 67.16 ± 7.48 years (*P* < 0.001). This may be due to the decline in hormone levels in postmenopausal women, who lack the protection of, for example, progesterone^36^ and oestrogen^37^.

Obesity has been shown to be associated with hyperuricaemia: firstly, obese individuals tend to have higher levels of uric acid compared to normal-weight individuals because of their higher urinary excretion and reduced clearance of uric acid^38^. Secondly, weight loss in obese individuals is accompanied by reduced uric acid levels and xanthine oxidoreductase (XOR) activity^39^; XOR is responsible for the breakdown of hypoxanthine and xanthine into uric acid. Finally, animal experiments have shown that the underlying mechanism of elevated uric acid in obese adipose tissue may be due to dysregulation of adipocytokines and chronic low-grade inflammation^40,41^.

A meta-analysis^42^ showed that sodium-glucose cotransporter 2 (SGLT-2) inhibitors might potentially prevent gout-related events in patients with type 2 diabetes mellitus, and recent studies^43,44^ have shown a reduction in blood uric acid levels in diabetic patients on glucose-lowering drugs. This may be related to the renal protective effects of hypoglycaemic agents^45–47^, such as SGLT-2 inhibitors, which not only promote anti-inflammatory and antifibrotic pathways, improve renal oxygenation, and reduce glomerular hypertension and hyperfiltration but also reduce the renal hypoxia characteristic of diabetes, thus exerting effects similar to those of β-blockers in the heart. However, the results of this survey showed that not taking glucose-lowering medications was negatively associated with hyperuricaemia in this type 2 diabetic population; however, the specific names of the glucose-lowering medications taken by this population were not available for this survey, and thus, further research is needed to confirm the results.

The mechanism of the blood pressure lowering effect on serum uric acid reduction is still under investigation. In a large trial of 10,617 hypertensive patients, therapeutic control of their blood pressure resulted in a significant reduction in the prevalence of hyperuricaemia^48^, similar to the present investigation. However, it has also been shown that appropriate systemic blood pressure control may lead to increased uric acid excretion through modulation of glomerular and tubular function, which in turn reduces serum uric acid and may ameliorate various forms of renal damage in the long term^49,50^.

Previous studies have shown that the kidneys eliminate 70% of uric acid daily^4^; therefore, the functional status of the kidneys also influences the development and progression of hyperuricaemia. Similarly, and similar to previous studies^51,52^, a decrease in the eGFR is indicative of a decrease in renal function, which can lead to serum uric acid retention and thus increase the risk of developing hyperuricaemia^53^. In the type 2 diabetic population, eGFR < 60 mL/(min × 1.73 m^2^) is generally defined as diabetic nephropathy (DN)^24^; therefore, approximately 7.44% (n = 613) of the patients in this population may have had DN, and further deterioration may lead to end-stage renal disease^54^. Additionally, some studies^55,56^ showed that type 2 diabetes mellitus combined with hyperuricaemia was associated with a higher risk of all-cause mortality and end-stage renal disease. Our model also suggests that when BUN is ≥ 7.5 mmol/L, this population is at increased risk of developing hyperuricaemia. For these reasons, emphasis should be placed on improving the screening and management of renal function in the type 2 diabetic population at an early stage.

When glycaemic control is poor in diabetic patients, uric acid levels are reduced owing to the permeability of glucose, causing increased excretion of urinary sugar, which in turn leads to competitive inhibition of uric acid reabsorption^57^, similar to the present findings. Recent studies have found that abnormal liver function is also a risk factor for the development of hyperuricaemia^58^, which may be related to the source of uric acid production. However, only elevated γ-glutamine transferase (GGT) was positively associated with hyperuricaemia in type 2 diabetic patients; thus, this investigation does not yet identify abnormal liver function as a risk factor for hyperuricaemia, and further studies are needed to support this.

Abnormalities in TC, TGs, HDL-C or LDL-C are generally diagnosed as dyslipidaemia, and dyslipidaemia is increasingly shown to be a risk factor for many diseases^59–61^. Previous studies^62,63^ have confirmed the positive correlation between TGs levels and hyperuricaemia, and Nakanishi et al.^64^ found that basal TGs remained an independent predictor of new-onset hyperuricaemia even when long-term medicated patients with diabetes mellitus were excluded, which is consistent with our study. Some studies have also attempted to explain the mechanism of elevated TGs and hyperuricaemia; as TGs rise, the production and utilisation of free fatty acids in the body increases and the catabolism of adenosine triphosphate is accelerated, leading to an increase in uric acid production^65^. Our study found a positive association between elevated LDL-C and hyperuricaemia, which may be related to the role of LDL-C by inducing vascular inflammation, atherogenesis, calcification and thrombosis^66^. In agreement with Xu et al.^67^, low HDL-C levels can trigger hyperuricaemia. HDL-C has anti-inflammatory, antioxidant and anti-apoptotic effects^68^, and it has also been found that HDL-C reduces inflammation induced by urate crystals, suggesting that HDL-C is involved in uric acid-induced inflammatory responses^69^.

## Clinical and public health potential

Our study identified a total of eleven factors affecting hyperuricaemia in the type 2 diabetes population, which could provide theoretical support in clinical decision-making and provide decision-making physicians with ideas for treating type 2 diabetes combined with hyperuricaemia. Meanwhile, in the health management of type 2 diabetes population, female type 2 diabetes patients should pay special attention to their uric acid level, and also strengthen the monitoring and management of risk factors such as abdominal obesity, elevated blood pressure, decreased liver and renal function, and dyslipidaemia, in order to the risk posed by type 2 diabetes mellitus combined with hyperuricaemia, which is of far-reaching significance for the prevention of progressive deterioration of the disease, the enhancement of the quality of life, and the reduction of medical costs.

## Strengths and Limitations

The strength of this study lies in its cross-sectional design to explore the risk factors for hyperuricaemia in a type 2 diabetes mellitus population with a large sample size, as well as the model based on logistic regression and ANN algorithms that were developed and fully validated. Our study also has many shortcomings. Firstly, the AUC value of our established ANN model is not outstanding and fails to reach the desired level. Secondly, although our study implemented strict inclusion and exclusion criteria, based on the nature of cross-sectional studies, the causal argument in determining hyperuricaemia remains unclear, and for this reason, further prospective studies are needed to validate it. Furthermore, it is difficult to control various biases in the survey, so that the truthfulness of some of the data is unconvincing. Finally, our study didn’t include enough variables to be explored, especially ignoring the effect of dietary factors on hyperuricaemia. Based on the above drawbacks, we will improve them in future studies to validate and refine the risk model.

## Conclusion

The ANN model built in this study based on eleven variables performed well and can provide theoretical support for clinical decision-making and self-care of type 2 diabetes mellitus patients to mitigate the harm caused by type 2 diabetes mellitus combined with hyperuricaemia.

## Data Availability

The datasets used and/or analysed during the current study available from the corresponding author on reasonable request.

## Abbreviations

ALT: Alanine aminotransferase
ANN: Artificial neural network
AUC: Area under the curve
BMI: Body mass index
BUN: Blood urea nitrogen
*CI*: Confidence interval
eGFR: Estimated glomerular filtration rate
FPG: Fasting glucose
GGT: γ-Glutamyl transpeptidase
HDL-C: High-density lipoprotein cholesterol
HUA: Hyperuricaemia
LDL-C: Low-density lipoprotein cholesterol
NLR: Negative likelihood ratio
NPV: Negative predictive value
PLR: Positive likelihood ratio
SCr: Serum creatinine
TBil: Total bilirubin
TC: Total cholesterol
TGs: Triglycerides
UA: Uric acid
R: Reference
